# Investigating Programmed Cell Death and Tumor Invasion in a Three-Dimensional (3D) Microfluidic Model of Glioblastoma

**DOI:** 10.3390/ijms21093162

**Published:** 2020-04-30

**Authors:** Ehsan Samiei, Amir Seyfoori, Brian Toyota, Saeid Ghavami, Mohsen Akbari

**Affiliations:** 1 Laboratory for Innovation in Microengineering (LiME), Department of Mechanical Engineering, University of Victoria, 3800 Finnerty Rd., Victoria, BC V8P 2C5, Canada; e.samiei@gmail.com (E.S.); am.seyfoori@gmail.com (A.S.); 2 Center for Advanced Materials and Related Technology (CAMTEC), University of Victoria, Victoria, BC V8W 2Y2, Canada; 3 Department of Surgery, Queens University, Kingston, ON K7L 2V7, Canada; briand.toyota@gmail.com; 4 Departments of Human Anatomy and Cell Science, Rady Faculty of Health Science, University of Manitoba, Winnipeg, MB R3E 0J9, Canada; saeid.ghavami@umanitoba.ca; 5 The Biology of Breathing Theme, Children’s Hospital Research Institute of Manitoba, University of Manitoba, Winnipeg, MB R3E 3P4, Canada

**Keywords:** tumor on a chip, glioblastoma, apoptosis, autophagy, cell phenotype, invasion

## Abstract

Glioblastoma multiforme (GBM) is a rapidly progressive and deadly form of brain tumor with a median survival rate of ~15 months. GBMs are hard to treat and significantly affect the patient’s physical and cognitive abilities and quality of life. Temozolomide (TMZ)—an alkylating agent that causes DNA damage—is the only chemotherapy choice for the treatment of GBM. However, TMZ also induces autophagy and causes tumor cell resistance and thus fails to improve the survival rate among patients. Here, we studied the drug-induced programmed cell death and invasion inhibition capacity of TMZ and a mevalonate cascade inhibitor, simvastatin (Simva), in a three-dimensional (3D) microfluidic model of GBM. We elucidate the role of autophagy in apoptotic cell death by comparing apoptosis in autophagy knockdown cells (Atg7 KD) against their scrambled counterparts. Our results show that the cells were significantly less sensitive to drugs in the 3D model as compared to monolayer culture systems. An immunofluorescence analysis confirmed that apoptosis is the mechanism of cell death in TMZ- and Simva-treated glioma cells. However, the induction of apoptosis in the 3D model is significantly lower than in monolayer cultures. We have also shown that autophagy inhibition (Atg7 KD) did not change TMZ and Simva-induced apoptosis in the 3D microfluidic model. Overall, for the first time in this study we have established the simultaneous detection of drug induced apoptosis and autophagy in a 3D microfluidic model of GBM. Our study presents a potential ex vivo platform for developing novel therapeutic strategies tailored toward disrupting key molecular pathways involved in programmed cell death and tumor invasion in glioblastoma.

## 1. Introduction

Glioblastoma multiforme (GBM) is a grade 4 astrocytoma, a brain cancer which accounts for 47.1% of malignant tumors in the central nervous system (CNS) [1,2]. GBM is highly malignant, and grows and spreads rapidly in the CNS [3]. Glioblastoma significantly affects the patient’s physical and cognitive abilities and quality of life, as the tumors are mostly located at the control center of thought, emotion, and movement. The current standard of care for newly diagnosed GBM is surgical resection to the extent feasible, followed by 6 weeks of radiotherapy with concurrent chemotherapy with temozolomide (TMZ). After the radiotherapy is finished, the monthly administration of TMZ is maintained for 6 months to 1 year [4]. Recently, it has been shown that statins could improve chemotherapy response in many cancers, including glioblastoma [5]. In a recent report, it has been demonstrated that lipophilic statin, simvastatin (Simva), which can cross the blood–brain barrier [6,7], significantly improved the survival of GBM patients who have been using TMZ [8].

Cell death occurs naturally or when stimulated through multiple pathways including apoptosis, autophagy, necrosis, senescence, and mitotic catastrophe [9]. In chemotherapy, apoptosis is the desired mechanism of cell death and is triggered through the alteration of the signaling mechanisms involved in cell death and proliferation [9]. Apoptosis and macroautophagy (hereafter autophagy), two main pathways of programmed cell death, are among the major mechanisms which are involved in chemotherapy response [9,10,11]. Caspases and PARP (poly-ADP ribose polymerase) cleavage are usually major hallmarks for apoptosis detection [12,13], while LC3 puncta and its co-localization with p62 is considered a reasonable marker for the detection of autophagy [10].

The molecular and genetic heterogeneity of gliomas result in a varied and often suboptimal response to treatment that is usually predicated on standard pathologic diagnoses. Research has elucidated many cellular and molecular mechanisms of tumor development, growth, and invasion, which has led to the identification of new cancer-specific molecular targets [14,15,16]. However, because most tumors are heterogeneous, a single drug regimen for patients with the same tumor type/histology is usually suboptimal [17]. As such, GBM is one of the deadliest cancers, with a 5-year survival rate of around 5.5% [3], and a median survival of only 15 months [18]. Given the growing availability of agents targeting different tumor compartments, combination therapy can be a promising approach that may lead to more robust or durable results. In particular, many experts agree that personalized cancer therapy based on the molecular characteristics of the tumor from an individual patient is a promising approach for the management of many types of cancer [19,20,21]. However, the primary challenge is the identification of drugs that are best administered together, which requires thorough biological evaluation supported by a profound understanding of the molecular mechanisms involved.

Pre-clinical research to delineate molecular mechanisms that drive cancer growth and progression or to determine the efficacy of experimental therapeutics is usually carried out in two-dimensional (2D) cancer cell cultures or in animal models. However, these models have had limited success because of the inability to recreate the complex interactions between cancer cells and the stroma environment in vivo. Although animal models have the advantage of providing the native three-dimensional (3D) microenvironment in which cancer cells reside, the challenges associated with interspecies variations, cost, and ethics remain major obstacles to the success of these models [22,23]. Therefore, there is a pressing need for developing bioengineered in vitro models that can mimic the 3D microenvironment and cytostructure of the brain tumor using human-derived cells and biomaterials.

Advancements in the synthesis and application of hydrogel biomaterials has made it possible to develop 3D cell constructs for tissue engineering and regenerative medicine [24]. It has been shown that hydrogel biomaterials used for 3D cell culture more or less mimic the extracellular matrix (ECM) of their native tissue [25]. Regulation of the intracellular signaling along with the mechanical cues from the hydrogels directs the cells to function more analogous to their native tissue [25,26,27]. These 3D cell culture models have been made in different configurations such as cell spheroids [28], bioprinted cell constructs [29], and microfluidic based organ-on-a-chip models [30]. Each of these 3D cell constructs has multiple applications, however the organ-on-a-chip models offer several advantages over others in terms of the flexibility and accuracy in the design of the model, and the capability of the controlled and directional delivery of nutrients, cytokines, and drugs to the cells [30]. These microfluidic and biomaterial-aided constructs have been widely used for developing tumor-on-a-chip models for simulating multiple phenomena such as tumor angiogenesis [31], tumor-vasculature interactions [32], invasion analysis [33], and tumor cell extravasation [34]. Although such tumor-on-a-chip models have been extensively used for the study of multiple cancer types, their application for brain cancer has been limited barely to a handful of studies [32,35]. Patient or stem cell-derived glioma cerebral organoids are 3D in vitro models of GBM which have recently been used for studying the biology of GBM [36,37]. They mimic the microenvironment of the tumor and are useful for the in vitro analysis of GBM biology. However, unlike microfluidic based models, there is no control over the cellular structure of such organoids. Additionally, microfluidic models allow the creation of perfusable conduits and the compartmentalization of the model, which are not possible with organoids. Nevertheless, for all cancer types the focus of tumor-on-a-chip studies has been mainly on mimicking the tumor microenvironment and cell/tissue functionality, while the application of such tumor-on-a-chip models for drug screening has been very limited [26,37]. Therefore, conducting a systematic drug screening study with the aid of tumor-on-a-chip models will make significant progress in the better understanding of brain cancer behavior and its response to chemotherapy treatments.

In this study, we report on the development of a bioengineered model of GBM in a microfluidic device and perform the first systematic drug screening analysis on the glioblastoma tumor on chip models. This study has three main objectives: (1) to study the difference in cell response to chemotherapy treatments between 2D cultures and the bioengineered glioblastoma model, (2) to study the role of autophagy in the programmed cell death of GBM cells, and (3) to evaluate cell invasion as a tool for quantifying drug efficacy. The glioblastoma-on-a-chip (GoC) model was comprised of a multi-compartment microfluidic device representing the tumor, stroma, and associated vascular system. Two common glioma cell lines, U251 and U87, were used for viability, invasion, and proof-of-principle drug studies. In particular, we used GoC to mimic the invasion of tumor cells into stroma in the presence and absence of cytokines. Invasion was used as a parameter for quantifying the effectiveness of the drugs, where the number of invaded cells was correlated to different concentrations of the drugs. Additionally, the mechanism of drug-induced cell death was studied by an immunofluorescence analysis of apoptosis and autophagy. We also used GBM cells with a knocked-down autophagy gene (Atg7 KD) to investigate the role of autophagy in TMZ and Simva-induced apoptosis in GoC and 2D culture models.

## 2. Results

### 2.1. Development and Analysis of Glioblastoma-on-a-Chip (GoC) Model

**Figure 1:**
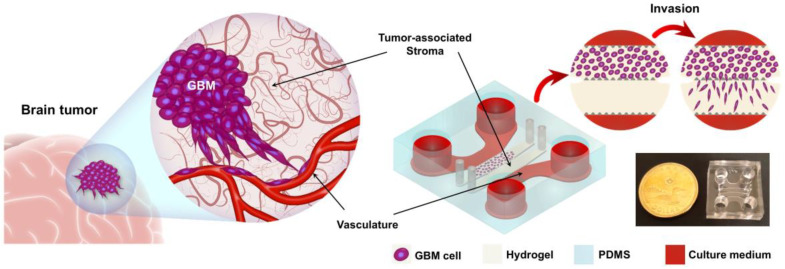
Concept of glioblastoma on chip (GoC). Schematic representation of glioblastoma multiforme (GBM) and the developed tumor-on-a-chip model. The GoC was comprised of a tumor and tumor-associated stroma compartments and side channels that delivered nutrients and drugs to the cells. The bottom right image shows the actual image of the fabricated model.

Glioblastoma is a highly aggressive brain tumor which infiltrates the brain rapidly. Here, we developed a bioengineered model of glioblastoma that mimicked the tumor microenvironment, tumor-associated stroma, and associated vasculature in a microfluidic device. Since the infiltrative behavior of GBM plays a significant role in the progression and expansion of tumor cells, the invasion of the cells has been thoroughly studied in GoC, considering the effects of chemotherapy agents on the invasiveness of the cells in the model. The main characteristic of GBM is that it diffuses through the brain [2]. The molecular cross-talk between the tumor cells, stroma tissue, and endothelial cells contributes to this behavior [38,39]. We recreated this configuration in a hydrogel-aided microfluidic platform comprised of four individually addressable parallel compartments made of polydimethylsiloxane (PDMS) (Figure 1). The two middle compartments were designed to mimic the tumor and the tumor-associated stroma layer. Two side channels were designed to supply cell media and drugs. Adjacent compartments were connected through the gaps between the posts. During the seeding process, interfacial tension prevented the leakage of the injected hydrogel to the side compartments [40]. The chip size was 25 mm.

**Figure 2:**
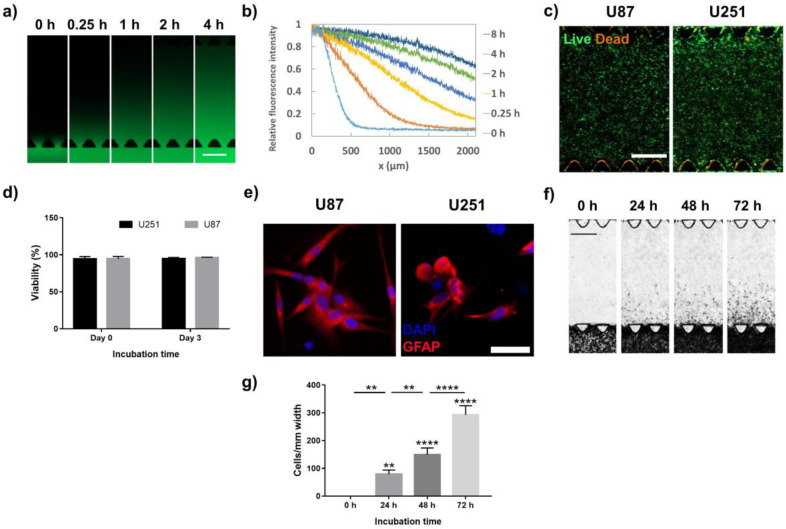
Characterization of GoC. (**a**) Image sequences showing the diffusion of fluorescein isothiocyanate-Dextran (FITC-dextran) (20 kDa) into the extracellular matrix (ECM)-like matrix (collagen concentration of 3 mg/mL). (**b**) The distribution of FITC-dextran along the collagen channel at different time points. (**c**) Live/dead staining of the U251 and U87 cells after 72 h of culture. (**d**) Quantitative viability of the U251 and U87 cells after 0 and 72 h of culture. (**e**) Immunofluorescence images of the U251 and U87 cells stained with glial fibrillary acidic protein (GFAP). (**f**) Bright field image sequences of the invasion of the U87 cells over 72 h. (**g**) Distribution profile of the invaded U87 cells across the channel at different time points. Data are expressed as mean ± standard deviation (*n* = 3). ** *p* < 0.01, **** *p* < 0.0001. In (**a**), (**c**), and (**f**), scale bars are 500 µm; in (**e**), scale bar is 50 µm.

Diffusion is the main mechanism of molecular transport within hydrogels. While the diffusion of small molecules occurs fast, larger molecules such as most growth factors diffuse at a much slower rate that can take up to a few hours to diffuse into the gel within millimeter scales. To study the molecular diffusion in our tumor model, the transport profile of a model large molecule, fluorescein isothiocyanate-Dextran (FITC-Dextran, 20 kDa), was measured in the GoC. The growth factors and other biomolecules and nutrients present in the culture medium had a variety of molecular weights, ranging from hundreds of Daltons to over 40 kDa (TGF-β1). FITC-Dextran with the molecular weight of 20 kDa was used here to show the overall diffusion behavior of large molecules in the hydrogel structure of the model. Other molecules in culture media have slower or faster diffusion rates depending on their molecular weight. The middle compartments of the model were loaded with cell-free collagen with a concentration of 3 mg/mL. In this study, we utilized collagen as the ECM component of the model as it has been widely used in previous studies for parenchymal infiltration and perivascular invasion in glioblastoma. To assure the functionality of the cells using collagen type 1, the cells were stained with glial fibrillary acidic protein (GFAP), a marker specific to astrocytes and glioma cells. However, in our future studies, we aim to use materials that closely mimic the ECM of native glioblastoma by including hyaluronic acid (HA), which is a main ECM component of the brain, laminin, and growth factors found in the tumor microenvironment. One of the side channels was filled with FITC-Dextran in Dulbecco’s phosphate buffered saline (DPBS) with a concentration of 2 mg/mL, and the other compartment was filled with DPBS to avoid the partial dehydration of the hydrogel. Live imaging was started immediately after loading the FITC-Dextran solution. Figure 2a shows the progression of the diffusion over 4 h. To quantify the diffusion, the fluorescent intensity was measured across the channel and was normalized with the intensity of the side channel. Figure 2b shows the profile of the FITC-Dextran concentration across the collagen compartment at different time points. Our results suggested that 4 h was required for a material with 20 kDa molecular mass to reach 50% of its original concentration at a distance of 2 mm from the boundary of the collagen compartment. 

We evaluated the ability of our GoC model to keep the glioblastoma cells viable and functional by analyzing the viability and phenotype of the cells cultured in the model. Since the incubation time for the experiments in this study was 72 h or less, the viability and phenotypic analyses were performed after 72 h of culture. For these tests, the models were made with a cell density of 1 × 10^6^ cells/mL without changing the cell media. Figure 2c shows fluorescent images of the U251 and U87 cells stained with L/D after 72 h of culture, indicating that the majority of the cells were viable. The viability of the cells immediately after seeding, and after 3 days of culture they showed a high level of viability (>95%), demonstrating that the culturing condition and materials used in the model did not induce cell death (Figure 2d).

The functionality of the cells in the tumor model was evaluated by the expression of the glial fibrillary acidic protein (GFAP) receptor, a specific marker for astrocytes and glioma cells [41,42]. The U251 and U87 cells in the tumor model were immunostained with GFAP after 72 h of incubation and imaged by confocal microscopy (Figure 2e). Both the U251 and U87 cells showed a positive GFAP expression, demonstrating the astrocyte phenotype of the cells used in our GoC model.

One important feature of the tumor-on-a-chip model developed in this study was the capability of analyzing the invasion of the tumor cells. To recapitulate the tumor invasion, one of the middle compartments was loaded with U87 cells with a density of 5 × 10^6^ cells/mL. The collagen concentration was 4 mg/mL. The adjacent compartment was filled with cell-free collagen with a concentration of 3 mg/mL to mimic the stroma tissue. This simplified model was used to quantify tumor infiltration into healthy brain tissue. The cells were allowed to grow for 72 h while bright field (BF) images were recorded every 24 h (Figure 2f). Our results showed that the number of cells and the invasion length in the invasion compartment increased continuously. Either the cell number or the invasion length can be used for the quantification of invasion. Here, we quantified the invasion by counting the number of cells in the stroma tissue. Figure 2g shows the progression of the number of invaded cells every 24 h. The cell counts were normalized with the width of the section that was used for quantification, and the results were presented per 1 mm width of the model.

### 2.2. Chemotherapy Treatment: Analysis of Programmed Cell Death and Invasion

The use of bioengineered tumor models has emerged as a powerful tool for evaluating existing and new drugs [23,28]. In particular, the potential of chip-based systems in producing a higher order cellular tissue organization and recapitulating disease formation and propagation as well as angiogenesis, inflammatory injury, and toxicity pathways in native tissues had led to their widespread use in disease modeling and drug screening. Moreover, since these miniaturized systems require small sample volumes (~nL), they can significantly reduce the costs associated with the drug development process. To show the feasibility of using GoC for drug studies, we performed a systematic viability study. The experiments involved exposing the cells to various doses of TMZ and Simva in GoC and in 2D culture systems. We paid special attention to three major mechanisms which are involved in the response of cancer cells to chemotherapy agents, including apoptosis, autophagy [10,43,44], and invasion. Initially, the cell viability and morphology of the cells that were treated with different concentrations of TMZ and Simva were studied and compared with the results of the conventional 2D culture systems. The induction of apoptosis in the cells was studied by the immunostaining of the cells with cleaved-PARP and cleaved-Caspase-3 as hallmark markers of apoptosis [45]. We also investigated the effect of the drugs on autophagy induction in a 3D GBM model by analyzing the autophagy hallmarks, SQSTM1p62 and LC3 puncta, and their co-localization by immunostaining the cells. The role of autophagy in cell death induction by chemotherapy agents in the 2D culture system and the GoC was studied by comparing the viability of U251 and U87 Atg7 KD with their corresponding scrambled cells. Finally, the invasion of the cells in the tumor model was evaluated by studying the effect of the chemotherapy drugs on the invasion rate of the cells.

Viability analyses were performed on U251 and U87. Cells with a density of 1 × 10^6^ cells/mL were encapsulated in collagen (3 mg/mL) in GoC and incubated for 24 h before chemotherapy treatment. For the 2D culture, the treatment was performed when the cells reached 40% confluency. The treatments with TMZ were performed at concentrations of 0, 100, 250, and 500 µM [46], and the Simva treatments were performed at concentrations of 0, 1, 5, and 10 µM [47]. A viability analysis was performed after 72 h of treatment and the results are shown in Figure S1a–d. It can be observed that for all cases, the viability of the cells decreased as the concentration of the drug was increased. Such behavior was statistically analyzed and the results are shown in Figure S1a–d, proving the dose-dependent pattern of the viability in both the GoC model and the 2D culture (*p* < 0.05–*p* < 0.0001). 

While TMZ and Simva induced cell death in both cell types, it was observed that the U251 cells were more sensitive to the treatments as compared to the U87 cells. This was further analyzed statically for the GoC viability results, and the statistical analysis results are shown in Figure S2. TMZ induces a significantly higher cell death in U251 compared to U87 cells in concentrations ≥ 100 µM. We have also observed the same pattern for the induction of cell death for Simva in concentrations ≥1 µM. For instance, as shown in Figure S2, 500 µM TMZ decreased the viability of U251 cells to 12%, whereas for U87 the viability was 45% (*p* < 0.0001). Simva with a 10 µM concentration also decreased the viability of the U251 and U87 cells to 7% and 49%, respectively (*p* < 0.0001). 

### 2.3. Drug Sensitivity Analysis of Cells in 3D GoC Model and 2D Cultures

**Figure 3:**
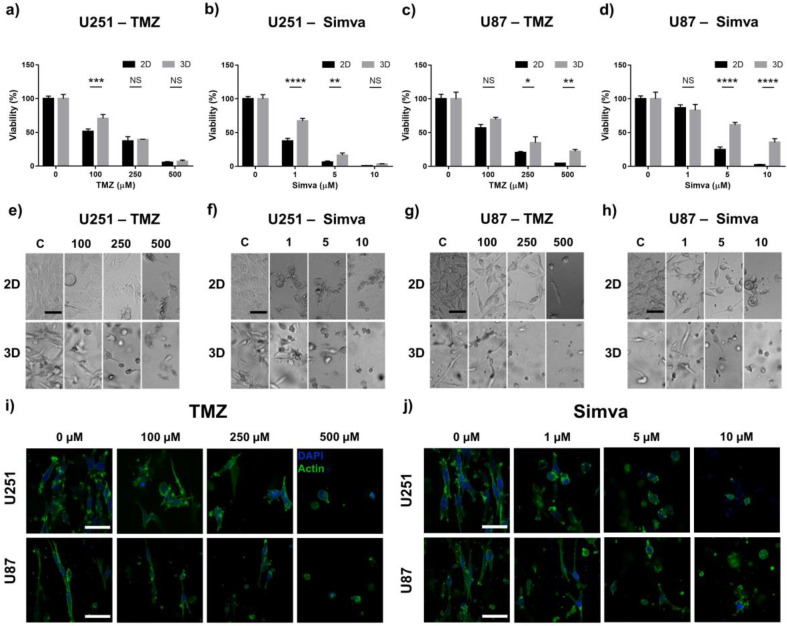
Drug sensitivity of the GoC and 2D culture systems. Viability and morphological analyses of the U251 and U87 cells after 72 h of treatment with different concentrations of temozolomide (TMZ) and Simva in the 2D culture and GoC. (**a**) Viability of the U251 cells treated with TMZ. (**b**) Viability of the U251 cells treated with Simva. (**c**) Viability of the U87 cells treated with TMZ. (**d**) Viability of the U87 cells treated with Simva, (**a**–**d**). Viability was calculated by live staining and normalizing each treatment with its control group. (**e**) Bright field images of the U251 cells treated with TMZ. (**f**) Bright field images of the U251 cells treated with Simva. (**g**) Bright field images of the U87 cells treated with TMZ. (**h**) Bright field images of the U87 cells treated with Simva. (**i**) Cytoskeleton of the U251 and U87 cells in the GoC treated with TMZ; actin (green), and DAPI (4′,6-diamidino-2-phenylindole) (blue). (**j**) Cytoskeleton of the U251 and U87 cells in the GoC treated with Simva; actin (green), and DAPI (blue). Data are expressed as mean ± standard deviation (*n* = 3). * *p* < 0.05, ** *p* < 0.01, *** *p* < 0.001, **** *p* < 0.0001. All scale bars are 50 µm.

Major barriers against the delivery of anticancer drugs in GBM are the blood–brain barrier and the diffusion limit caused by tumor-associated stroma and the ECM of the tumor. A low drug penetration can result in a low drug concentration around target cancer cells, which can cause an incomplete response to the drug. Two-dimensional cell monolayers fail to recapitulate the diffusion barrier caused by the tumor ECM and the stroma layer. However, the GoC platform reported in this work mimics the 3D structure of the tumor microenvironment and diffusive transport of drugs and nutrients in vitro. In is worth noting that modeling the blood–brain barrier was not in the scope of this work and will be considered in future studies. We performed a drug toxicity study by exposing the cells in the GoC to TMZ (0–500 µM) and Simva (0–10 µM) and compared their viability with 2D cultures (Figure 3a–d). The cell viability for the U251 cells was significantly higher in the GoC model as compared to the 2D culture (*p* < 0.05–*p* < 0.0001). For U251, at the upper limit of drug concentrations (500 µM TMZ and 10 µM Simva) on the other hand, the majority of the cells in both models were dead with no significant difference between the 2D and GoC results. For the U87 cells, at 250 µM and 500 µM TMZ, as well as at 5 µM and 10 µM Simva, the viability of the cells in the GoC was significantly higher than in the 2D culture (*p* < 0.05–*p* < 0.0001). 

Besides the cytotoxic effect of the TMZ and Simva on the GBM cells, the effect of these drugs on the morphology and cytoskelton of the cells was also investigated. As shown in Figure 3e–h, in the control group both the U251 and U87 cells were elongated, with a more mesenchymal-like morphology. As the concentration of the drugs increased, the cells became less elongated and showed an epithelial-like morphology, and for the highest doses of the drugs, the cells looked fairly round. To further confirm this, we stained the F-actin cytoskeleton of the cells that were treated with different concentrations of TMZ and Simva (Figure 3i,j). Results indicate that both the TMZ and Simva significantly affected the cytoskeleton of the cells, in a way that increasing the drug concentration decreased the expression of the network of F-actin filaments. Such a lowering effect on the cell cytoskeletal structure affected the morphology of the cells, resulting in a round and smaller cell structure as the drug concentration increased.

### 2.4. Drug-Induced Apoptosis in 2D Culture System and GoC 

**Figure 4:**
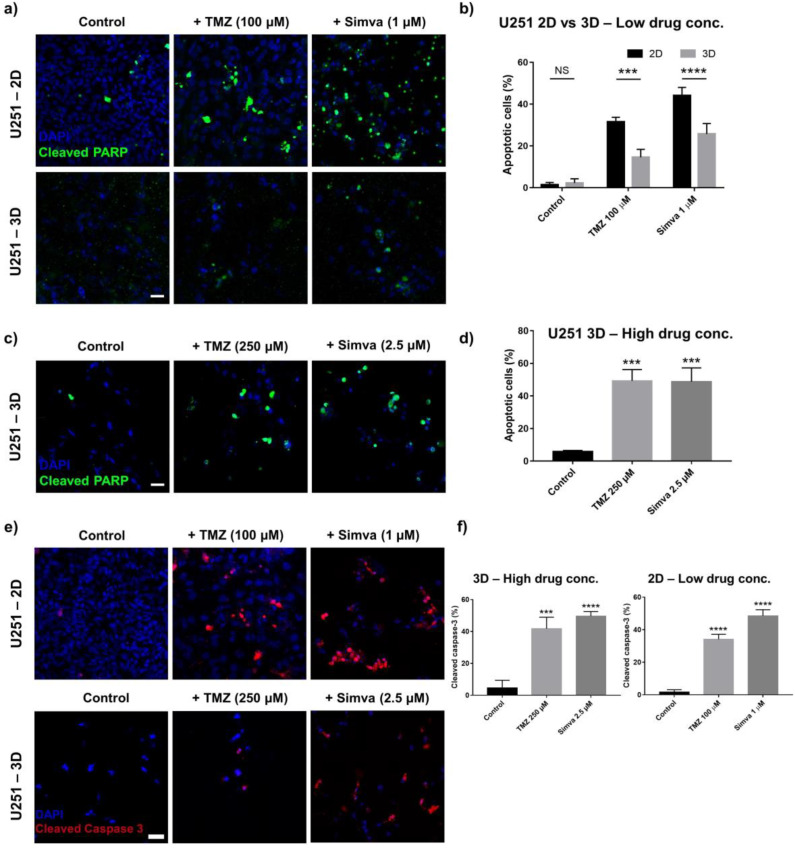
Drug-induced apoptosis in glioma cells in the 2D culture and GoC. (**a**) Immunofluorescence staining of the U251 cells with cleaved PARP after 72 h of treatment with TMZ (100 µM) and Simva (1 µM) in the 2D culture and GoC model. (**b**) Comparison of the expression level of the cleaved PARP (percentage of the number of cleaved PARP to nuclei) between the 2D and GoC models for low drug concentrations (TMZ 100 µM, Simva 1 µM). (**c**) Immunofluorescence staining of the U251 cells with cleaved PARP after 72 h of treatment with TMZ (250 µM) and Simva (2.5 µM) in the GoC model. (**d**) Expression level of the cleaved PARP in the U251 and U87 cells in the GoC model for high drug concentrations (TMZ 250 µM, Simva 2.5 and 5 µM). (**e**) Immunofluorescence staining of the U251 cells with cleaved Caspase-3 after 60 h of treatment with TMZ (100 µM for 2D and 250 µM for microfluidic model) and Simva (1 µM for 2D and 2.5 µM for GoC model). (**f**) Expression level of cleaved Caspase 3 (percentage of the number of cleaved Caspase 3 to nuclei) in both the 2D and 3D models. Data are expressed as mean ± standard deviation (*n* = 3). *** *p* < 0.001, **** *p* < 0.0001. All scale bars are 50 µm.

In cancer treatment and specifically in chemotherapy, it is desired that tumor cells undergo a programmed cell death (apoptosis), in which the cell death occurs through the disintegration of the cell components and engulfment of them by other cells [9]. Other cell death mechanisms, specifically necrosis, involve undesired side effects, including inflammatory effects [48]. To study the mechanism of the action of the drugs studied in this work, the treated cells were immune-stained for cleaved-PARP after 72 h of culture. The cleavage of the PARP occurs when the cells undergo apoptosis. First, the responses of the U251 cells to treatments with 100 µM of TMZ and 1 µM of Simva in the 2D culture system and the GoC were compared (Figure 4a). The significantly higher PARP cleavage for the treated samples as compared to the control (untreated) indicated that the TMZ and Simva induced apoptosis in the cells (*p* < 0.001, and *p* < 0.0001 for TMZ and Simva, respectively). However, the 2D culture system exhibited a higher population of apoptotic cells as compared to the GoC results (*p* < 0.001, and *p* < 0.0001 for TMZ and Simva, respectively). The level of cleaved PARP was quantified by measuring the ratio of the apoptotic cells (with PARP cleavage) to the total number of cells in each sample (Figure 4b). In the 2D culture, the population of apoptotic cells that were treated with the TMZ and Simva were about 30% and 45% of the total cells, respectively, whereas the population of apoptotic cells was significantly lower in the GoC (about 15% and 25% for TMZ and Simva, respectively). This confirms the viability results in the previous section, showing that the cells grown in the biomimetic model were less sensitive to the treatments. The level of apoptosis in the U251 cells was studied in the GoC for a higher concentration of the drugs (TMZ 250 µM and Simva 2.5 µM) to investigate whether apoptosis is concentration-dependent. Figure 4c shows the immunostaining images and Figure 4d illustrates the quantified results, in which the U251 shows over 45% apoptosis for both drugs.

To further confirm the occurrence of drug-induced apoptosis in the GBM cells, the cleavage of caspase-3 in the U251 cells was studied using immunofluorescence staining. Caspase-3 is an executioner of apoptosis and its activation induces the proteolytic cleavage of multiple proteins including PARP [49]. The cleavage of the caspase-3 occurs in the stage prior to the PARP, and therefore we immunostained the U251 cells with cleaved caspase-3 after 60 h of treatment (12 h shorter than the incubation time for cleaved PARP). The immunofluorescence images and the cleavage level of this marker in the U251 cells are shown in Figure 4e,f, respectively. Positive cleaved caspase-3 can be observed in the cells treated with the drugs in both configurations, confirming the activation of the apoptosis pathway due to the treatment with TMZ and Simva.

### 2.5. Effect of TMZ and Simva in the Autophagy Pathway and Its Effect in Their Apoptosis Induction

**Figure 5:**
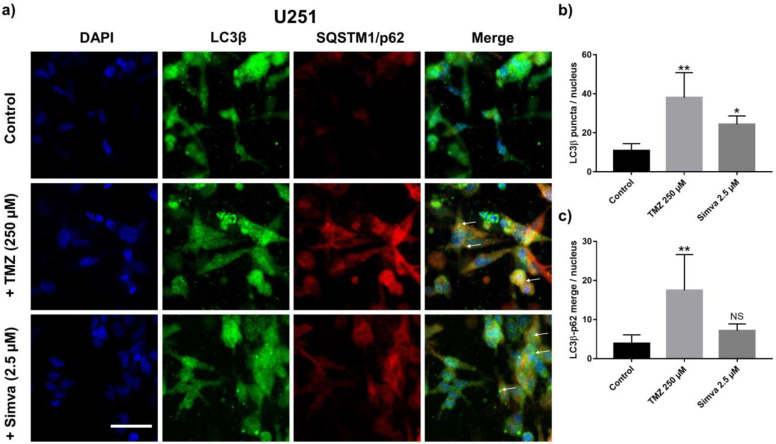
Drug-induced autophagy in the cells in the GoC. (**a**) Immunofluorescence staining of the U251 cells with LC3β (green) and SQSTM1/p62 (red) after 72 h of treatment with TMZ (250 µM) and Simva (2.5 µM) in the 3D model. (**b**) Expression level of LC3β (ratio of the number of LC3β puncta to the number of nuclei). (**c**) Ratio of the number of co-localized LC3β puncta and p62 to the number of nuclei. Data are expressed as mean ± standard deviation (*n* = 3). * *p* < 0.05, ** *p* < 0.01. Scale bar is 50 µm.

Autophagy is a cellular response to stress in which damaged organelles and the misfolded proteins of cells are collected in autophagosomes and subsequently delivered to the lysosome for degradation [10]. Depending on the level of cellular stress, autophagy could lead to cell survival or, in severe situations, it will lead to cell death [10]. Autophagy can be triggered by several mechanisms, such as starvation, hypoxia, and chemotherapy [50]. Here, the changes in the autophagy pathway in GBM cells due to the treatment with TMZ and Simva were studied in the GoC model. We stained the U251 cells with LC3β and p62, two main markers of autophagosome formation and autophagy flux, after 72 h of treatment with TMZ (250 µM) and Simva (2.5 µM). The immunofluorescence images for the U251 cells are shown in Figure 5a, in which DAPI (blue), LC3β (green), p62 (red), and merged images are shown in different panels. For the samples treated with TMZ and Simva, there was an increase in the formation of LC3β puncta, which is a marker for the formation of autophagosomes, while such a high number of LC3 puncta was not observed in the control group. The LC3β punctuation (pointed with arrows in Figure 5a) was quantified by counting the average number of LC3β puncta per nucleus (Figure 5b). The graph shows that the treatments significantly increased the number of LC3β puncta compared to the control group (*p* < 0.01 for TMZ and *p* < 0.05 for Simva). Besides the formation of LC3β puncta, their co-localization with p62 is an indicator of autophagy flux (delivery of autophagosome to lysosomes), which is pointed out by arrows in the TMZ treatment [10]. The number of LC3 puncta co-localized with p62 was quantified for the control and treatment groups, which is shown in Figure 5c. The co-localization in TMZ group was significantly higher than in the control group (*p* < 0.01), while the difference between the Simva and control was not significant. This shows that the TMZ induced autophagy flux in the cells, while Simva did not show such a level of co-localization, revealing that Simva inhibited autophagy flux in the U251 cells even though it induced an increase in LC3 puncta. 

**Figure 6:**
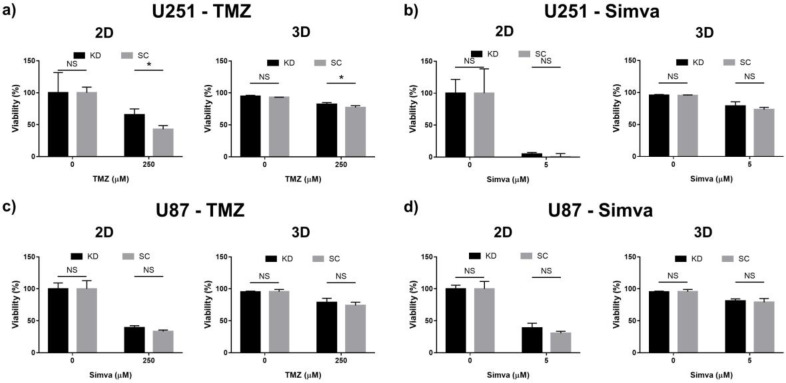
Analysis of the effect of autophagy on the viability of the U87 and U251 cells in response to treatment with TMZ and Simva in the 2D culture and GoC. For each case, the Atg7 knocked down (U251 KD and U87 KD) and scrambled (U251 SC and U87 SC) cells have been compared. The 2D and 3D viability were calculated using PrestoBlue and live/dead staining assays, respectively. (**a**) U25—TMZ, (**b**) U251—Simva, (**c**) U87—TMZ, (**d**) U87—Simva. Data are expressed as mean ± standard deviation (*n* = 3), * *p* < 0.05.

It is well known that autophagy can either support or inhibit apoptosis in a cellular system [51,52]. Atg7 is one of the key regulators of autophagy which is upstream to autophagosome formation [53,54]. Therefore, the knockdown of Atg7 affects the entire autophagy process and is a key parameter to investigate the autophagy pathway. To investigate how autophagy was involved in the regulation of TMZ- and Simva-induced cell death, the viability of the Atg7 knockdown (KD) and their scrambled (SC) counterparts were evaluated when the cells were treated with TMZ and Simva. In brief, the U87 and U251 cells were treated with TMZ (250 µM) and Simva (5 µM) and their viability was compared to that of the untreated cells (control). Our results (Figure 6) suggest that Atg7 KD did not affect the Simva-induced cell death, whereas for TMZ the viability of the KD and SC cells was only different for the U251 cells. This observation confirmed that the Atg7-dependent autophagy pathway is not involved in Simva-induced cell death in both U87 and U251 cells, while TMZ-induced cell death is Atg7-dependent in U251 cells.

### 2.6. Effect of TMZ and Simva on Cell Invasion in GoC

**Figure 7:**
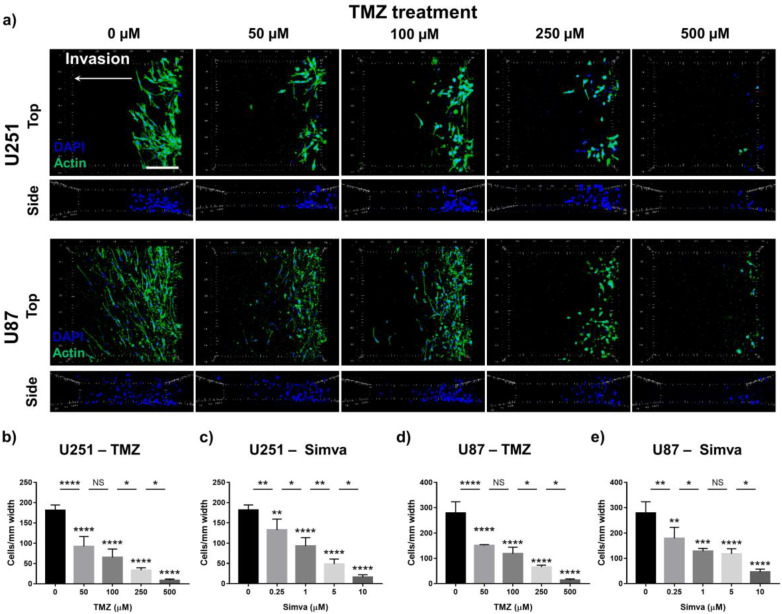
Effect of the drug treatment on tumor invasion in the GoC. (**a**) Z-stacked DAPI/actin immunofluorescence images of the U251 and U87 cells invading to the side compartment under treatment with different concentrations of temozolomide (0–500 µM). Number of invaded cells per mm width of the chip for the U251 and U87 cells treated with TMZ (0–500 µM) and Simva (0–10 µM): (**b**) U251 cells, TMZ; (**c**) U251 cells, Simva; (**d**) U87 cells, TMZ; and (**e**) U87 cells, Simva. Data are expressed as mean ± standard deviation (*n* = 3). * *p* < 0.05, ** *p* < 0.01, *** *p* < 0.001, **** *p* < 0.0001. Scale bar is 200 µm.

One of the main advantages that our GoC model offers over the conventional 2D culture plates is its ability to model and quantify cell invasion. Here, invasion was introduced as a parameter for evaluating the efficacy of the drugs. For this purpose, tumor models with U251 and U87 cells were prepared, as explained in the materials and methods section, and treated with TMZ (0, 50, 100, 250 and 500 µM) and Simva (0, 0.25, 1, 5 and 10 µM) for 72 h. The Z-stack images of the U251 and U87 cells treated with TMZ are shown in Figure 7a, illustrating the top and side views of the invasion profile of the cells in the invasion compartment. The side view images of the nuclei illustrate that the cells were uniformly distributed across the height of the chip. The bright field images of the invaded cells are shown in Figure S3.

The top view images show the invasion pattern of the cells and their morphology (actin). The number of infiltrated cells in the stromal region was counted from the BF images. Our results show that the glioma invasion was reduced in a dose-dependent manner, such that increasing the drug concentration decreased the number and travel length of the invaded cells. The invasion behaviors of the U251 and U87 cells were different; the U251 cells invaded in close proximity of each other and traveled over a shorter distance, while the U87 cells were more elongated and invaded over a longer distance. We quantified the invasion for both cell types and both drugs based on the number of invaded cells per 1 mm width of the tumor model, and the results are shown in Figure 7b–e. For all conditions, the invasion showed a sensitive response to different doses of the drugs, following a similar trend to the viability results shown in Figure 3a–d. The viability analysis (Figure S1) showed that 50 µM of TMZ and 0.25 µM of Simva are non-toxic for the cells in the 3D model, while the invasion of the cells treated with such drug doses was significantly suppressed (Figure 7). This showed that not only did cell death decrease the invasion, but also that the TMZ and Simva treatments suppressed the invasiveness of the cells. Besides the decrease in the number of invaded cells caused by increasing the drug dose, the morphology of the invaded cells also was significantly affected by increasing the concentration of the drugs. The cells in the control condition had a highly protrusive morphology, and treating them with the chemotherapy agents induced a less protrusive and rounder morphology in a dose-dependent manner.

**Figure 8:**
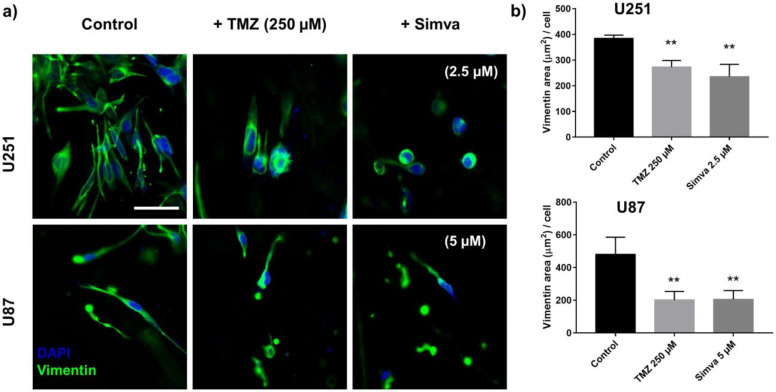
Effect of drugs on tumor invasiveness. (**a**) Immunofluorescence staining of the U251 and U87 cells with vimentin after 72 h of treatment with TMZ (250 µM) and Simva (2.5 µM for U251 and 5 µM for U87) in the microfluidic tumor model. (**b**) Expression level of vimentin (average area coverage by vimentin per nuclei). Data are expressed as mean ± standard deviation (*n* = 3). ** *p* < 0.01. Scale bar is 50 µm.

It was shown that TMZ and Simva decreased the invasion of the U251 and U87 cells in the GoC model. To further confirm the effect of these drugs on the invasiveness of the cells, the expression of vimentin, a marker indicative of cellular phenotype and invasiveness, in the U251 and U87 cells was studied after 72 h of treatment with the TMZ (250 µM) and Simva (2.5 µM for U251 and 5 µM for U87). Figure 8a illustrates the immunofluorescence images of the cells stained with vimentin. The expression level of vimentin was quantified by measuring the average area of vimentin per nucleus (Figure S4), and the results are shown in Figure 8b. The graphs show that the level of vimentin in the control condition was significantly higher than in the treated condition (*p* < 0.01). This shows that the cells in the control condition showed a more mesenchyme-like behavior, compared to the cells treated with TMZ and Simva, which expressed more epithelial-like behavior, suggesting that treatment with TMZ and Simva reduces the invasiveness of the cells. Such a suppression in invasiveness, along with the reduction in the number of viable cells in the treated cells, reduced the number of invaded cells (Figure 7), suggesting invasion as a sensitive parameter for the analysis of the effects of chemotherapy treatments on GBM.

## 3. Discussion

The low survival rate in patients diagnosed with GBM in comparison with other cancer types is known to be due to its high malignancy, heterogeneity, and invasive behavior [3,18,55]. This situation does not leave enough time for clinical studies to thoroughly understand the patient-specific physiological behavior of GBM, and more importantly, perform systematic clinical trials for personalized drug development. Animal tests are typically costly and time consuming, with a relatively low relevance to human GBM [56]. In vitro tests also have been limited to monolayer cell culture plates, which fail to mimic the physiological condition of the disease in vivo, resulting in the unrealistic response of the cells to the chemotherapy agents [25,57]. Especially, the analysis of parameters such as the invasiveness or morphological conditions of the tumor cells has been highly limited due to the lack of a 3D structure with the presence of an extracellular matrix (ECM) [58]. Such limitations have resulted in the lack of a proper therapy procedure, and consequently a low survival rate for the patients.

The emergence of microfluidic-based tissue or disease models with the aid of biomaterials has made a significant advancement in developing in vitro models with a higher relevance to the physiological conditions of the native tissues or diseases [24]. Several models have been developed using this technology, with the main focus on the complexity and phenotypic behavior of the tissue [30]. However, there is still a lack of systematic study on drug screening using such 3D tissue models. Developing in vitro models which better mimic the physiological conditions of the disease will significantly improve the drug screening results, reducing the total pharmacological costs and speeding up the drug development process. With this goal, we developed a GBM tumor model comprised of a multichannel microfluidic device and hydrogel scaffolds that mimic the 3D structure of the tumor. Tumor cells can be embedded within the collagen hydrogel at different cell densities. Our model allows for homogeneous cell distribution for viability and pathway analyses, and non-homogeneous cell distribution for modeling invasion. Since the goal of developing the model was to conduct systematic drug screening analyses, a co-culture of multiple cells was not used in order to avoid complications in performing the viability and pathway analyses. 

The capabilities of our microfluidic tumor model were shown by performing several analyses, which are shown in Figure 2. First, the mechanism of material transport was shown by studying the diffusion rate of FITC-dextran, as a relatively large molecule, through the 3D structure of the model. Similarly to the in vivo conditions, in 3D scaffolds the molecules in the culture medium will diffuse through the structure of the scaffold to be delivered to the cells, while in 2D models the molecules are always readily available to the cells. This would have had a significant impact on the response of the cells to the chemotherapy agents. The model maintained the tumor cells viable and functional, showing over 95% viability after 3 days of culture and expressing GFAP, which is the main marker of glioma cells. Then, the capability of the developed platform in modeling invasion was illustrated by designing the model such that the tumor compartment was placed adjacent to a cell-free hydrogel compartment, allowing the cells to invade in a quantifiable manner. This feature allowed for quantifying tumor invasion under different culture conditions and treatments. 

In the next step, drug screening on glioblastoma was performed using our GoC model along with a 2D culture for comparison. TMZ, an FDA (Food and Drug Administration)-approved drug for GBM [59], and Simva, a cholesterol-lowering drug which is used independently or in many cases combined with other drugs [60], were used as the chemotherapy agents for our studies. Culturing U251 and U87 cells in both the 2D and GoC model revealed a significant difference in the morphology of the cells, such that in the 2D the cells spread over the culture plate with a large exposed surface area, while they had a tubular morphology in the tumor model. Along with the difference in the mechanisms of material transport, such morphological differences altered the exposure of the cells to the chemotherapy drugs. The viability analyses in Figure 3 revealed that the GBM cell viability decreased due to the treatment with both TMZ and Simva in a dose-dependent manner. However, the cells in the GoC model had a significantly lower response (higher viability) to the treatments in comparison to the 2D culture. Monitoring the morphological changes in the cells due to the chemotherapy treatments in the GoC model was also a powerful tool for the drug screening analysis, which was performed using both bright field images and the immunostaining of the F-actin in the cells. Increasing the drug concentration changed the morphology of the cells from a protruded tubular form to a round shape. This confirmed the previous findings regarding the effect of chemotherapy agents on GBM cell morphology [61] and epithelial to mesenchymal transition [62].

Based on our observations, several mechanisms could be responsible for such a significant difference in the 2D and GoC viability results. While the cells in the 2D culture were surrounded with the drug-loaded culture medium, in the microfluidic model the drug had to diffuse through the hydrogel, and as a result, it experienced a higher resistance while being delivered to the cells [26]. Another source of discrepancy was the difference in the morphologies of the cells. As shown in Figure 3e–h, the cells in the 2D culture spread over the culture plate and exposed a high surface area to the medium. In the 3D culture, on the other hand, the cells were tubular and elongated, exposing a significantly lower surface area to the medium [63]. This difference in the surface area of the cells in the 2D and 3D cultures could possibly have caused a lower delivery of the drugs to the cells in the 3D model.

It has been shown that TMZ induced apoptosis in GBM cells in vivo and in 2D and spheroid models [64,65]. Here, the mechanism of cell death in our hydrogel-based GoC model was studied by immunostaining the treated and untreated cells with apoptosis markers, cleaved PARP, and cleaved caspase-3, as shown in Figure 4. We had previously confirmed the induction of apoptosis in GBM cells by TMZ and Simva treatments in 2D culture using immunostaining, Western Blot, and flow cytometry [53]. In this study, we only used the immunostaining technique for the analysis of apoptosis. It was observed that for U251, the treatment with TMZ and Simva induced apoptosis in both the 2D and microfluidic configurations. However, the level of apoptosis was significantly higher in the 2D culture, confirming the trend in the viability results in which the cells in the microfluidic model showed a higher resistance to the chemotherapy treatments. A higher level of apoptosis was observed for the U251 and U87 cells (around 45% and 35%, respectively) in the microfluidic model when higher drug concentrations (TMZ 250 µM, and Simva 2.5/5 µM for U87/U251) were used.

Autophagy is a cellular mechanism which is closely related to the regulation of programmed cell death, where its induction/inhibition could either support or inhibit cell death [3,66]. Through this process, the denatured proteins and damaged intracellular organelles are collected within the cells by the autophagosomes. Subsequently, the autophagosomes are digested when they merge with lysosomes, forming autophagolysosomes. We performed immunostaining for LC3 and p62 to evaluate the autophagy flux after the treatment with TMZ and Simva in our GoC model (Figure 5). We had previously confirmed the induction of autophagy in GBM cells by TMZ and Simva in 2D culture using immunostaining and Western Blot [53]. In this study, we only used the immunostaining technique for the analysis of autophagy. Comparing the immunofluorescence results of the control and treatment conditions revealed that TMZ induces an autophagy flux in the cells, which is confirmed via the formation of LC3β puncta (autophagosome formation) and their co-localization with p62 (autophagosomes and lysosomes merge, which indicates the delivery of autophagosme to lysosomes). Previous findings had also shown the induction of autophagy by TMZ [67,68], which is in line with our findings. On the other hand, in our GoC model Simva inhibited the autophagy flux, as the LC3β puncta did not co-localize with p62. This was in line with several studies which used different statins as the chemotherapy agent. Zhang et al. [69] showed that 10 µM of lovastatin inhibited the autophagy flux in C2C12 cells through declining protein kinase D activity. Su et al. also showed the inhibition of autophagy flux by simvastatin in heart cells, which was studied by the LC3II/LC3I ratio and the expression level of p62 [70]. In contrast, some other studies identified autophagy flux due to treatment with pepstatin, which was based on the observation of LC3 II [71,72]. Such controversial observations could be due to different cell types and/or the difference in the dose and incubation time of the drug.

In addition to the formation of autophagosomes and their merging with lysosomes, autophagy provides adenosine triphosphate (ATP) for ATP-dependent mechanisms, including apoptosis. To study this, the chemotherapy treatment of the cells with the Atg7 KD gene was compared with their corresponding SC cells (Figure 6). It was observed that for majority of the treatment conditions, the viabilities of the KD and SC cells were not significantly different, and only for the U251 cells treated with TMZ, the KD cells had a higher viability. This showed that autophagy has a minor effect on the induction of apoptosis in GBM cells which were treated with TMZ and Simva in our GoC model, which was in agreement with some previous in vitro and clinical findings [67,73]. Therefore, we concluded that autophagy is a parallel mechanism to apoptosis in our model. 

In the last step, invasion was introduced as a sensitive parameter of the study for drug screening. The invasion of the GBM cells was studied in the microfluidic tumor model treated with TMZ and Simva, and the results were quantified and are shown in Figure 7. It was observed that the number of invaded cells decreased by increasing the drug concentration, which is an indication of the effect of these chemotherapy agents on the invasiveness of the cells. The morphology of the invading cells was also highly indicative of the negative effect of the chemotherapy drugs on the invasiveness of the cells, since the untreated invading cells were highly protrusive, showing mesenchymal-like behavior. Increasing the drug concentration reduced such behavior, resulting in a more epithelial-like behavior. Previous studies have shown that the invasiveness of cancer cells is highly reflected in the expression level of vimentin in the cells [74]. It has been shown that invasive cells with a more protrusive morphology that express high mesenchymal markers express a higher level of vimentin in comparison with cells with epithelial behavior [74,75]. We studied this in our GoC model by immunostaining the treated and control cells with vimentin, and the results showed a suppression in the expression level of vimentin (Figure 8) for the cells treated with TMZ and Simva in comparison with the control group. This confirmed the negative effect of the chemotherapy drugs on the invasiveness of the GBM cells. Our findings revealed that invasion analysis in such engineered 3D platforms is an informative and useful analysis method for drug screening applications in a dose-dependent manner, which could be used along with other analyses. 

## 4. Materials and Methods

### 4.1. Materials

A polydimethylsiloxane (PDMS) elastomer kit was purchased from Ellsworth Adhesives Co. (Germantown, WI, USA); SU-8 100 and its developer were purchased from Kayaku Advanced Materials, Inc. (Westborough, MA, USA); a high glucose Dulbecco’s modified eagle medium (DMEM) with L-glutamine, penicillin streptomycin (Pen Strep, 10,000 units/mL penicillin and 10,000 µg/mL streptomycin), fetal bovine serum (FBS), 0.5% trypsin-EDTA, temozolomide, simvastatin, live/dead staining viability kit, puromycin dihydrochloride, fluorescein isothiocyanate-Dextran (FITC-Dextran), Dulbecco’s phosphate buffered saline (DPBS), and poly-D-lysine were purchased from Millipore Sigma (Oakville, ON, Canada). All the immunofluorescence primary antibodies, SQSTM1/p62 (D5L7G) Mouse mAb, LC3B (D11) XP^®^ Rabbit mAb, cleaved PARP (Asp214) (D64E10) XP^®^ Rabbit mAb (Alexa Fluor^®^ 488 Conjugate), cleaved Caspase-3 (Asp175) (D3E9) Rabbit mAb (Alexa Fluor^®^ 647 Conjugate), GFAP (GA5) Mouse mAb #3670, and Vimentin (D21H3) XP^®^ Rabbit mAb (Alexa Fluor^®^ 488 Conjugate), were purchased from Cell Signaling Technology (Danvers, MA, USA). The secondary antibodies with conjugated fluorescence (Alexa Fluor^®^ 488 AffiniPure Donkey Anti-Rabbit IgG, Alexa Fluor^®^ 647 AffiniPure Donkey Anti-Mouse IgG), and IgG-free bovine serum albumin (BSA) were purchased from Jackson ImmunoResearch Inc. (West Grove, PA, USA). The DAPI (4’,6-Diamidino-2-Phenylindole, Dihydrochloride), Alexa Fluor™ 488 Phalloidin, and PrestoBlue cell viability reagent were purchased from Thermo Fisher Scientific (Waltham, MA, USA). The polybrene, puromycin, Atg7 shRNA lentiviral article, and shRNA control lentiviral particle-A were purchased from Santa Cruz Biotechnology Inc. (Dallas, TX, USA). The 10× phosphate buffered saline (PBS), 0.5 N NaOH and bovine collagen type 1 (10 mg/mL) were purchased from Advanced BioMatrix Inc. (San Diego, CA, USA). The formaldehyde 37% solution was purchased from VWR (Mississauga, ON, Canada). The triton X-100 was purchased from Bio Basic Canada Inc. (Toronto, ON, Canada). All other reagents were of analytical grade.

### 4.2. Microfluidic Device Fabrication

The microfluidic device was comprised of four individually addressable parallel compartments made in PDMS. The two middle compartments were designed to mimic the tumor and the tumor-associated stroma layer. The two side channels were designed to supply cell media and drugs. All the adjacent compartments were separated using an array of small posts with ~ 100 µm gaps between them. These gaps allowed for the diffusion of molecules and liquids between different compartments. The microfluidic devices were fabricated by bonding polydimethylsiloxane (PDMS) microchannel patterns on a coverslip. To fabricate the microchannels within the PDMS piece, an SU-8 master mold was fabricated by the photolithography method using SU-8 100 on a microscope slide, with the fabrication parameters adjusted to achieve a thickness of 200 µm for the SU-8 layer. The PDMS base elastomer was mixed with its curing agent in a ratio of 1:10, degassed in a desiccator, poured on the SU-8 master mold, and cured on a hotplate for 1.5–2 h at 80 °C. Subsequently, the PDMS layer was gently detached from the mold and 1 mm and 5 mm diameter openings were punched through them using biopsy punches for hydrogel chambers and media channels, respectively. After the plasma treatment, the PDMS chip was bonded to the cover slip and baked on a hotplate for 2 h at 80 °C. To remove debris or particles and to sterilize the chips, they were incubated in 70% ethanol for 10 min, rinsed with pure ethanol and dried, and finally were exposed to ultra-violet (UV) light for 30 min. To ensure the complete removal of ethanol and retain the highest hydrophobicity of the devices, they were baked at 80 °C for 4 h in a sterile vented container. To enhance the attachment of the hydrogel to the microfluidic device, the surfaces of the microchannels were coated with poly-D-lysine (PDL). Briefly, a 1 mg/mL solution of PDL in distilled water was injected in the channels and incubated at 37 °C for 1 h. Then, the PDL was removed and the device was washed with distilled water 3 times. To retain the hydrophobicity of the devices, they were incubated at 80 °C for 4 h.

### 4.3. Monolayer Cell Culture

Human-derived glioma cell lines, U251 (Creative Bioarray, CSC-6321W) and U87 (ATCC^®^ HTB-14™), which have been derived from malignant glioblastoma tumors, were used for the studies. A humidified incubator (Thermofisher Scientific) at 37 °C supplied with 5% CO_2_ was used for the cell culture. The cells were cultured in T75 flasks in a medium containing high glucose DMEM, 10% FBS, and 1% Pen/Strep. The culture medium was replaced every 24–48 h until cells reached 70–80% confluence. At this confluence, the culture medium was removed and the cells were rinsed with DPBS and then detached by incubating them with 0.25% typsin-EDTA for 3–5 min. The trypsin was neutralized by adding the culture medium and then the suspension was centrifuged at 200 G for 5 min. After removing the supernatant, the cell palette was re-suspended in the culture medium and counted using a hemocytometer. 

We used the Atg7 knockdown (KD) and scrambled (SC) U251 and U87 cells that were prepared in our previous studies [51,53,76]. Briefly, the U87 and U251 cells were cultured in 6-well plates in DMEM supplemented with 10% FBS until they reached 40% confluence. The cells were then treated with polybrene (10 µg/mL, Santa Cruz; sc-134220) in FBS-free DMEM for 1 h, and subsequently were transfected with shRNA Lentiviral Particles for the Atg7 and scrambled control (negative control, with the same nucleotide composition which includes no specific sequence). Both contained the puromycin resistance codon (Santa Cruz; sc-41447-V, *APG7* shRNA (h) Lentiviral Particles); the transfection was performed for 12 h using 3 and 6 multiplicity of infections (MOI), then the media was replaced for 24 h for recovery. The cells with incorporated shRNA plasmid were cultured in a puromycin dihydrochloride complete DMEM medium (4 µg/mL). The expression of Atg7 was tested in selected cells from both the shRNA-transfected and scrambled control cells using Western blotting [76].

For all the 2D cell experiments, the cells were seeded in 96- or 12-well plates and incubated for 1 or 2 days to reach 30% and 40% confluency for the U251 and U87 cells, respectively. The treatment was then started by replacing the medium with the medium containing the drug. The treatments were performed for 72 h without replacing the medium.

### 4.4. 3D Cell Culture

The tumor compartment was composed of cell-loaded collagen type I (4 mg/mL), and the stroma tissue was formed from cell-free collagen at a concentration of 3 mg/mL. For the studies that involved invasion, the tumor compartment was seeded first and cured, then the invasion compartment was injected and cured. After the middle compartments were loaded with the cells and hydrogel and cured, the culture media was added to the side compartments, as explained in the materials and methods section. The biological and biophysical characteristics of the model were studied in the following sections. The 3D cell culture was performed by encapsulating the cells in bovine collagen type 1 at two different concentrations of 3 mg/mL and 4 mg/mL depending on the cell density. For the viability and immunostaining studies, a final cell density of 1 × 10^6^ cells/mL was used, for which the collagen concentration was set to 3 mg/mL. For the invasion studies, a cell density of 5 × 10^6^ cells/mL was used, with a collagen concentration of 4 mg/mL. The higher concentration of collagen provided a higher stiffness, preventing the contraction of the scaffold due to the mechanical force by cells at a higher population. 

To prepare the hydrogel/cell suspension, first the pH and ionic concentrations of the collagen solution were adjusted by adding 10 × PBS and 0.5 normal NaOH to the stock solution of collagen with a ratio of 1:1:8. Following that, the cell suspension with an adjusted cell density was mixed with the collagen solution to achieve the desired collagen concentration and final cell density [40]. For the invasion studies, a solution of collagen without cells was prepared with a concentration of 3 mg/mL. In the entire seeding process, the solutions were kept on ice to avoid pre-crosslinking of the collagen. The collagen/cell suspension was injected in the microfluidic channel and then incubated in the humidified incubator at 37 °C for 45 min until the collagen was crosslinked. For the invasion studies, once the cell compartment was crosslinked, the cell-free collagen solution was injected into the invasion compartment next to the cell compartment and incubated for 45 min until it was crosslinked. After curing the hydrogels, the culture medium was added to one side of the channels and incubated at 37 °C for a few minutes so that the culture media slowly filled the media compartment with the aid of gravity and the wetting effect of the condensed droplets. For the viability and immunostaining studies, the cells were cultured overnight and the treatment was started the day after. For the invasion studies, the treatment was started immediately after seeding.

### 4.5. Cell Viability Analysis

For the 3D culture (GoC), the cell viability was evaluated by fluorescence microscopy of the samples stained with the live/dead kit [43]. The staining solution was prepared according to the supplier’s protocol. Briefly, 200 µL of 1 μM calcein AM (live) and 4 μM ethidium homodimer-1 (dead) were added to the wells and incubated at room temperature in the dark for 2 h. Fluorescent images were then taken for the analysis of the cell viability. For the comparison of the GoC results, the viability was calculated by dividing the number of live cells by the total number of live and dead cells.

A 2D cell viability analysis was performed either using PrestoBlue reagent or live/dead staining. PrestoBlue was used for the cases where only the 2D viability results were compared with each other. In brief, the 10 × stock solution of PrestoBlue was diluted in the culture medium with a ratio of 1:10. The medium was removed from the 2D cell culture and the diluted PrestoBlue solution was added and incubated for 30 min in the incubator. Immediately after incubation, 200 µL of each sample was transferred to a 96-well plate and the fluorescent intensity was measured using a plate reader at 560 nm and 590 nm excitation and emission wavelengths. Blank measurements were subtracted from the cell samples and the viability was calculated by normalizing the treatment results with the control sample.

For the comparison of the cytotoxicity of the chemotherapy agents on the 2D and GoC models, live staining was performed to assure the similarity of the viability assay for both cases. The cells in both models were treated with different concentrations of the drugs (0, 100, 250, and 500 µM for TMZ and 0, 1, 5, and 10 µM for Simva). After 72 h of treatment, the cells were stained with calcein AM (live) in both models and the number of viable cells was counted. The viability was calculated by normalizing the number of viable cells for each treatment with the number of viable cells in the control group in the same category.

### 4.6. Immunofluorescence Staining 

An analysis of different pathways was performed by immunostaining the cells with relevant biomarkers [43]. First, the cells were fixed by adding a 3.7% solution of formaldehyde in DPBS to the wells (2D) or microfluidic device (3D) and incubating at room temperature for 20–30 min. Then, the solution of formaldehyde was removed and the sample was washed with DPBS 3 times, each time incubating for 5 min. Following that, the samples were permeablized and blocked at 4 °C with a blocking buffer containing 5% normal serum and 0.3% triton-x 100 in DPBS (2 h for 2D and overnight for 3D culture). Then, the blocking buffer was removed and a solution of marker-specific primary antibodies was added to the wells and incubated overnight (both 2D and 3D) at 4 °C in the dark. To prepare the solution of primary antibodies, 1% BSA and 0.3% triton × 100 in DPBS was used as the dilution buffer, and the antibodies were diluted in the solution with the ratio recommended by the supplier. The primary antibodies either were or were not conjugated with the fluorescent tag. The conjugated antibodies (cleaved PARP, cleaved Caspase 3, and Vimentin) did not require a secondary antibody conjugation. For the samples incubated with non-conjugated antibodies (GFAP, LC3, and p62), the solution of primary antibodies was removed, the samples were washed 3 times with DPBS, and the solution of fluorescent-conjugated secondary antibodies was added to the wells and incubated for 2–4 h at 4 °C in the dark (3D). In the next step, for both the conjugated and non-conjugated cases the antibody solution was removed and the solution of DAPI was added to the wells and incubated for 30 min for the 2D culture and 2 h for the 3D culture at room temperature in the dark to stain the nuclei. The DAPI was diluted in DPBS with the concentration recommended by the supplier. Finally, the DAPI solution was removed and the samples were washed with DPBS 3 times, each time incubating for 5 min. Imaging was performed immediately after the staining, and DPBS was added to the wells to avoid the dehydration of the samples.

Cytoskeleton actin staining was performed using Alexa Fluor™ 488 Phalloidin, according to the supplier’s protocol. Briefly, the cells were fixed as explained above and then permeablized using a solution of 0.3% triton × 100 in DPBS for 30 min at room temperature. Then, the triton solution was removed from the microfluidic wells and a solution of actin was added to the wells and incubated at room temperature for 2–3 h in the dark. The actin solution was prepared by diluting the stock solution of Alexa Fluor™ 488 Phalloidin in DPBS containing 1% BSA and 0.3% triton × 100 with the ratio suggested by the supplier. Following that, the DAPI staining and washing was performed as explained above.

### 4.7. Imaging

The bright field (BF) and L/D fluorescent images were obtained using an Axio Observer ZEISS microscope (ZEISS, Oberkochen, Germany) with 2.5× and 10× magnification objectives. The biomarker immunostaining images were obtained using a ZEISS confocal microscope (Zeiss LSM880, ZEISS, Oberkochen, Germany) with 20× and 50× magnification objectives.

### 4.8. Statistical Analysis

All the experiments were repeated at least three times, and the values were calculated by averaging the results of the replicates. The error bars show ± standard deviation from the average values. A significance analysis was performed by a two-tailed t-test or one-way or two-way ANOVA using a GraphPad Prism. 

## 5. Conclusions

The drug development process involves the testing thousands of compounds on cells grown in dishes in animal models and through multiple phases of clinical trials before a single drug can be brought into the market. These studies can take several years and can cost billions of dollars to complete. Meanwhile, innumerable animal lives are lost and the process often fails to predict human responses to drugs because animal models do not accurately mimic human pathophysiology. This work aims to provide a quick and economical method as a drug screening assay to study the effect of different treatment strategies on programmed cell death and glioma invasion. The model reported herein was comprised of a multi-compartment microfluidic device with embedded cell-loaded and cell-free hydrogel scaffolds, which allowed the performance of drug screening assays including viability and pathway analyses. We used this platform to quantify drug-induced apoptosis, investigate the crosstalk between drug-induced autophagy and apoptosis, and evaluate the effect of drugs on tumor invasion. We showed that the drug response in the GoC model was significantly lower than in conventional 2D culture systems. Moreover, the cell morphology was significantly different in our 3D model from the 2D cultures. We showed that apoptosis was the main mechanism of cell death when the cells were treated with chemotherapy agents. The level of apoptosis in the microfluidic model was lower than in the 2D culture system. Immunostaining with LC3β and p62 showed that TMZ induced autophagy flux while Simva inhibited it in the GBM cells. To study whether autophagy inhibits or promotes cell death, we performed a viability analysis using KD (Atg7 knockdown) and SC (scrambled) cells, and it was observed that autophagy had a minor effect on inducing cell death by TMZ treatment. Finally, we studied the effect of TMZ and Simva on the invasion of the cells and showed that an increase in the drug dose decreased the number of invaded cells. Immunostaining of the cells using vimentin also confirmed the reduction in the invasiveness of the cells due to the chemotherapy treatments. The significant difference in invasion results revealed that invasion could be considered as a sensitive and reliable parameter for studying the response of the tumor cells to chemotherapy treatments. The reported chip-based platform holds great promise for developing new therapeutic agents tailored to individual patients and is a step towards personalizing the treatments. However, we acknowledge that stromal cells play critical roles in the microenvironment and the response of glioblastoma to therapy. As such, our future studies will be focused on increasing the complexity of our model by adding relevant stroma cells and forming a functional blood–brain barrier in our model. 

## Supplementary Materials

Supplementary materials can be found at https://www.mdpi.com/1422-0067/21/9/3162/s1.

## References

[1] Ostrom Q.T., Gittleman H., Xu J., Kromer C., Wolinsky Y., Kruchko C., Barnholtz-Sloan J.S. (2016). CBTRUS statistical report: Primary brain and other central nervous system tumors diagnosed in the United States in 2009–2013. Neuro Oncol..

[2] Holland E.C. (2000). Glioblastoma multiforme: The terminator. Proc. Natl. Acad. Sci. USA.

[3] Hombach-Klonisch S., Mehrpour M., Shojaei S., Harlos C., Pitz M., Hamai A., Siemianowicz K., Likus W., Wiechec E., Toyota B.D. (2018). Glioblastoma and chemoresistance to alkylating agents: Involvement of apoptosis, autophagy, and unfolded protein response. Pharmacol. Ther..

[4] Stummer W., Pichlmeier U., Meinel T., Wiestler O.D., Zanella F., Reulen H.J., Yang I., Aghi M.K., Ohgaki H., Hau P. (2005). Radiotherapy plus concomitant and adjuvant temozolomide for glioblastoma. Neuropathology.

[5] Nielsen S.F., Nordestgaard B.G., Bojesen S.E. (2012). Statin Use and Reduced Cancer-Related Mortality. N. Engl. J. Med..

[6] Wassif C.A., Kratz L., Sparks S.E., Wheeler C., Bianconi S., Gropman A., Calis K.A., Kelley R.I., Tierney E., Porter F.D. (2017). A placebo-controlled trial of simvastatin therapy in Smith-Lemli-Opitz syndrome. Genet. Med..

[7] Patel Y.T., Jacus M.O., Davis A.D., Boulos N., Turner D.C., Vuppala P.K., Freeman B.B., Gilbertson R.J., Stewart C.F. (2016). Simvastatin Hydroxy Acid Fails to Attain Sufficient Central Nervous System Tumor Exposure to Achieve a Cytotoxic Effect: Results of a Preclinical Cerebral Microdialysis Study. Drug Metab. Dispos..

[8] International T., Epidemiology C., Gaist D. (2014). Statin use and survival following glioblastoma multiforme. Cancer Epidemiol..

[9] Ricci M.S., Zong W. (2006). Chemotherapeutic Approaches for Targeting Cell Death Pathways. Oncologist.

[10] Klionsky D.J., Abdelmohsen K., Abe A., Abedin M.J., Abeliovich H., Arozena A.A., Adachi H., Adams C.M., Adams P.D., Adeli K. (2016). Guidelines for the use and interpretation of assays for monitoring autophagy (3rd edition). Autophagy.

[11] Elmore S. (2007). Apoptosis: A Review of Programmed Cell Death. Toxicol. Pathol..

[12] Cohen G.M. (1997). Caspases: The executioners of apoptosis. Biochem. J..

[13] Chaitanya G.V., Alexander J.S., Babu P.P. (2010). PARP-1 cleavage fragments: Signatures of cell-death proteases in neurodegeneration. Cell Commun. Signal..

[14] Workman P., Collins I. (2007). Corrigendum: New approaches to molecular cancer therapeutics. Nat. Chem. Biol..

[15] Vogelstein B., Kinzler K.W. (2004). Cancer genes and the pathways they control. Nat. Med..

[16] William W.N., Heymach J.V., Kim E.S., Lippman S.M. (2009). Molecular targets for cancer chemoprevention. Nat. Rev. Drug Discov..

[17] Ferté C., André F., Soria J.C. (2010). Molecular circuits of solid tumors: Prognostic and predictive tools for bedside use. Nat. Rev. Clin. Oncol..

[18] Alifieris C., Trafalis D.T. (2015). Glioblastoma multiforme: Pathogenesis and treatment. Pharmacol. Ther..

[19] Wistuba I.I., Gelovani J.G., Jacoby J.J., Davis S.E., Herbst R.S. (2011). Methodological and practical challenges for personalized cancer therapies. Nat. Rev. Clin. Oncol..

[20] Junttila M.R., De Sauvage F.J. (2013). Influence of tumour micro-environment heterogeneity on therapeutic response. Nature.

[21] Bartlett R., Everett W., Lim S., Natasha G., Loizidou M., Jell G., Tan A., Seifalian A.M. (2014). Personalized in vitro cancer modeling—Fantasy or reality?. Transl. Oncol..

[22] Nyga A., Cheema U., Loizidou M. (2011). 3D tumour models: Novel in vitro approaches to cancer studies. J. Cell Commun. Signal..

[23] Valente K.P., Khetani S., Kolahchi A.R., Sanati-Nezhad A., Suleman A., Akbari M. (2017). Microfluidic technologies for anticancer drug studies. Drug Discov. Today.

[24] Annabi N., Tamayol A., Uquillas J.A., Akbari M., Bertassoni L.E., Cha C., Camci-Unal G., Dokmeci M.R., Peppas N.A., Khademhosseini A. (2014). 25th anniversary article: Rational design and applications of hydrogels in regenerative medicine. Adv. Mater..

[25] Tibbitt M.W., Anseth K.S. (2009). Hydrogels as extracellular matrix mimics for 3D cell culture. Biotechnol. Bioeng..

[26] Breslin S., O’Driscoll L. (2013). Three-dimensional cell culture: The missing link in drug discovery. Drug Discov. Today.

[27] Tekin H., Simmons S., Cummings B., Gao L., Adiconis X., Hession C.C., Ghoshal A., Dionne D., Choudhury S.R., Yesilyurt V. (2018). Effects of 3D culturing conditions on the transcriptomic profile of stem-cell-derived neurons. Nat. Biomed. Eng..

[28] Seyfoori A., Samiei E., Godau B., Jalili N., Rahmanian M., Farahmand L., Majidzadeh K., Akbari M. (2018). Self-Filling Microwell Arrays (SFMAs) for Tumor Spheroid Formation. Lab Chip.

[29] Murphy S.V., Atala A. (2014). 3D bioprinting of tissues and organs. Nat. Biotechnol..

[30] Bhatia S.N., Ingber D.E. (2014). Microfluidic organs-on-chips. Nat. Biotechnol..

[31] Bischel L.L., Young E.W.K., Mader B.R., Beebe D.J. (2013). Tubeless microfluidic angiogenesis assay with three-dimensional endothelial-lined microvessels. Biomaterials.

[32] Truong D., Fiorelli R., Barrientos E.S., Melendez E.L., Sanai N., Mehta S., Nikkhah M. (2019). A three-dimensional (3D) organotypic microfluidic model for glioma stem cells—Vascular interactions. Biomaterials.

[33] Truong D., Puleo J., Llave A., Mouneimne G., Kamm R.D., Nikkhah M. (2016). Breast cancer cell invasion into a three dimensional tumor-stroma microenvironment. Sci. Rep..

[34] Zervantonakisa I.K., Hughes-Alfor S.K., Charest J.L., Condeelis J.S., Gertler F.B., Kamm R.D. (2012). Three-dimensional microfluidic model for tumor cell intravasation and endothelial barrier function. Proc. Natl. Acad. Sci. USA.

[35] Ayuso J.M., Monge R., Martínez-González A., Virumbrales-Muñoz M., Llamazares G.A., Berganzo J., Hernández-Laín A., Santolaria J., Doblaré M., Hubert C. (2017). Glioblastoma on a microfluidic chip: Generating pseudopalisades and enhancing aggressiveness through blood vessel obstruction events. Neuro Oncol..

[36] Ogawa J., Pao G.M., Shokhirev M.N., Verma I.M. (2018). Glioblastoma Model Using Human Cerebral Organoids. Cell Rep..

[37] Linkous A., Balamatsias D., Snuderl M., Edwards L., Miyaguchi K., Milner T., Reich B., Cohen-Gould L., Storaska A., Nakayama Y. (2019). Modeling Patient-Derived Glioblastoma with Cerebral Organoids. Cell Rep..

[38] Kenig S., Alonso M.B.D., Mueller M.M., Lah T.T. (2010). Glioblastoma and endothelial cells cross-talk, mediated by SDF-1, enhances tumour invasion and endothelial proliferation by increasing expression of cathepsins B, S, and MMP-9. Cancer Lett..

[39] Demuth T., Berens M.E. (2004). Molecular mechanisms of glioma cell migration and invasion. J. Neurooncol..

[40] Shin Y., Han S., Jeon J.S., Yamamoto K., Zervantonakis I.K., Sudo R., Kamm R.D., Chung S. (2012). Microfluidic assay for simultaneous culture of multiple cell types on surfaces or within hydrogels. Nat. Protoc..

[41] Velpula K.K., Dasari V.R., Tsung A.J., Dzung H., Rao J.S. (2011). Cord blood stem cells revert glioma stem cell EMT by down regulating transcriptional activation of Sox2 and Twist1. Oncotarget.

[42] Yun J.H., Park S.J., Jo A., Kang J.L., Jou I. (2011). Caveolin-1 is involved in reactive oxygen species-induced SHP-2 activation in astrocytes. Exp. Mol. Med..

[43] Moghadam A.R., da Silva Rosa S.C., Samiei E., Alizadeh J., Field J., Kawalec P., Thliveris J., Akbari M., Ghavami S., Gordon J.W. (2018). Autophagy modulates temozolomide-induced cell death in alveolar Rhabdomyosarcoma cells. Cell Death Discov..

[44] Emami A., Shojaei S., da Silva Rosa S.C., Aghaei M., Samiei E., Vosoughi A.R., Kalantari F., Kawalec P., Thliveris J., Sharma P. (2019). Mechanisms of simvastatin myotoxicity: The role of autophagy flux inhibition. Eur. J. Pharmacol..

[45] Ghavami S., Hashemi M., Ande S.R., Yeganeh B., Xiao W., Eshraghi M., Bus C.J., Kadkhoda K., Wiechec E., Halayko A.J. (2009). Apoptosis and cancer: Mutations within caspase genes. J. Med. Genet..

[46] Lin C.J., Lee C.C., Shih Y.L., Lin T.Y., Wang S.H., Lin Y.F., Shih C.M. (2012). Resveratrol enhances the therapeutic effect of temozolomide against malignant glioma in vitro and in vivo by inhibiting autophagy. Free Radic. Biol. Med..

[47] Yanae M., Tsubaki M., Satou T., Itoh T., Imano M., Yamazoe Y., Nishida S. (2011). Statin-induced apoptosis via the suppression of ERK1/2 and Akt activation by inhibition of the geranylgeranyl-pyrophosphate biosynthesis in glioblastoma. J. Exp. Clin. Cancer Res..

[48] Proskuryakov S.Y., Konoplyannikov A.G., Gabai V.L. (2003). Necrosis: A specific form of programmed cell death?. Exp. Cell Res..

[49] Porter A.G., Ja R.U. (1999). Emerging roles of caspase-3 in apoptosis. Cell Death Differ..

[50] Lin L., Baehrecke E.H. (2015). Autophagy, cell death, and cancer. Mol. Cell. Oncol..

[51] Ghavami S., Eshragi M., Ande S.R., Chazin W.J., Klonisch T., Halayko A.J., McNeill K.D., Hashemi M., Kerkhoff C., Los M. (2010). S100A8/A9 induces autophagy and apoptosis via ROS-mediated cross-talk between mitochondria and lysosomes that involves BNIP3. Cell Res..

[52] Song S., Tan J., Miao Y., Li M., Zhang Q. (2017). Crosstalk of autophagy and apoptosis: Involvement of the dual role of autophagy under ER stress. J. Cell. Physiol..

[53] Shojaei S., Koleini N., Samiei E., Aghaei M., Cole L.K., Alizadeh J., Islam M.I., Vosoughi A.R., Albokashy M., Butterfield Y. (2020). Simvastatin increases temozolomide-induced cell death by targeting the fusion of autophagosomes and lysosomes. FEBS J..

[54] Alizadeh J., Glogowska A., Thliveris J., Kalantari F., Shojaei S., Hombach-Klonisch S., Klonisch T., Ghavami S. (2018). Autophagy modulates transforming growth factor beta 1 induced epithelial to mesenchymal transition in non-small cell lung cancer cells. Biochim. Biophys. Acta Mol. Cell Res..

[55] Stupp R., Hegi M.E., Mason W.P., van den Bent M.J., Taphoorn M.J.B., Janzer R.C., Ludwin S.K., Allgeier A., Fisher B., Belanger K. (2009). Effects of radiotherapy with concomitant and adjuvant temozolomide versus radiotherapy alone on survival in glioblastoma in a randomised phase III study: 5-year analysis of the EORTC-NCIC trial. Lancet Oncol..

[56] McGonigle P., Ruggeri B. (2014). Animal models of human disease: Challenges in enabling translation. Biochem. Pharmacol..

[57] Imamura Y., Mukohara T., Shimono Y., Funakoshi Y., Chayahara N., Toyoda M., Kiyota N., Takao S., Kono S., Nakatsura T. (2015). Comparison of 2D- and 3D-culture models as drug-testing platforms in breast cancer. Oncol. Rep..

[58] Taubenberger A.V., Bray L.J., Haller B., Shaposhnykov A., Binner M., Freudenberg U., Guck J., Werner C. (2016). 3D extracellular matrix interactions modulate tumour cell growth, invasion and angiogenesis in engineered tumour microenvironments. Acta Biomater..

[59] Wen P.Y., Reardon D.A. (2016). Progress in glioma diagnosis, classification and treatment. Nat. Rev. Neurol..

[60] Alizadeh J., Zeki A.A., Mirzaei N., Tewary S., Moghadam A.R., Glogowska A., Nagakannan P., Eftekharpour E., Wiechec E., Gordon J.W. (2017). Mevalonate Cascade Inhibition by Simvastatin Induces the Intrinsic Apoptosis Pathway via Depletion of Isoprenoids in Tumor Cells. Sci. Rep..

[61] Pan Q., Wang X.Y.H. (2012). Chemoresistance to Temozolomide in Human Glioma Cell Line U251 is Associated with Increased Activity of O 6 -methylguanine- DNA Methyltransferase and Can be Overcome by Metronomic Temozolomide Regimen. Cell Biochem. Biophys..

[62] Kubelt C., Hattermann K., Sebens S., Mehdorn H.M., Held-feindt J. (2015). Epithelial-to-mesenchymal transition in paired human primary and recurrent glioblastomas. Int. J. Oncol..

[63] Yamada K.M., Cukierman E. (2007). Modeling Tissue Morphogenesis and Cancer in 3D. Cell.

[64] Roos W.P., Batista L.F.Z., Naumann S.C., Wick W., Weller M., Menck C.F.M., Kaina B. (2007). Apoptosis in malignant glioma cells triggered by the temozolomide-induced DNA lesion O 6 -methylguanine. Oncogene.

[65] Pawlak E., Damasceno R., Arnold H., Terzis A.J., Allee R. (2003). Temozolomide induces apoptosis and senescence in glioma cells cultured as multicellular spheroids. Br. J. Cancer.

[66] Zhang J., Stevens M.F., Bradshaw T.D. (2012). Temozolomide: Mechanisms of Action, Repair and Resistance. Curr. Mol. Pharmacol..

[67] Kanzawa T., Germano I.M., Komata T., Ito H., Kondo Y., Kondo S. (2004). Role of autophagy in temozolomide-induced cytotoxicity for malignant glioma cells. Cell Death Differ..

[68] Würstle S., Schneider F., Ringel F., Gempt J., Lämmer F., Delbridge C., Wu W.E.I., Schlegel J., Neuropathology D., München T.U. (2017). Temozolomide induces autophagy in primary and established glioblastoma cells in an EGFR independent manner. Oncol. Lett..

[69] Zhang P., Verity M.A., Reue K. (2014). Lipin-1 regulates autophagy clearance and intersects with statin drug effects in skeletal muscle. Cell Metab..

[70] Su F., Shi M., Zhang J., Zheng Q., Zhang D., Zhang W., Wang H., Li X. (2018). Simvastatin Protects Heart from Pressure Overload Injury by Inhibiting Excessive Autophagy. Int. J. Med. Sci..

[71] Loos B., Du Toit A., Hofmeyr J.H.S. (2014). Defining and measuring autophagosome flux—Concept and reality. Autophagy.

[72] Mizushima N., Yoshimori T., Levine B. (2010). Methods in Mammalian Autophagy Research. Cell.

[73] Yan Y., Xu Z., Dai S., Qian L., Sun L., Gong Z. (2016). Targeting autophagy to sensitive glioma to temozolomide treatment. J. Exp. Clin. Cancer Res..

[74] Mendez M.G., Kojima S., Goldman R.D. (2010). Vimentin induces changes in cell shape, motility, and adhesion during the epithelial to mesenchymal transition. FASEB J..

[75] Nguemgo Kouam P., Rezniczek G.A., Kochanneck A., Priesch-Grzeszkowiak B., Hero T., Adamietz I.A., Bühler H. (2018). Robo1 and vimentin regulate radiation- induced motility of human glioblastoma cells. PLoS ONE.

[76] Ghavami S., Yeganeh B., Stelmack G.L., Kashani H.H., Sharma P., Cunnington R., Rattan S., Bathe K., Klonisch T., Dixon I.M.C. (2012). Apoptosis, autophagy and ER stress in mevalonate cascade inhibition-induced cell death of human atrial fibroblasts. Cell Death Dis..

