# Supplementary material for: Investigating Programmed Cell Death and Tumor Invasion in a Three-Dimensional (3D) Microfluidic Model of Glioblastoma
Source: Int J Mol Sci. 2020 Apr 30;21(9):3162. doi: 10.3390/ijms21093162 (PMC7246580; doi:10.3390/ijms21093162)
Supplement: Supplementary file 1 [file ijms-21-03162-s001.pdf]

Supplementary Information

Investigating programmed cell death and tumor invasion in a three-dimensional (3D) microfluidic model of glioblastoma

Ehsan Samiei[1, 2], Amir Seyfoori[1, 2], Brian Toyota[3], Saeid Ghavami[4, 5], Mohsen Akbari[1, 2,*]

[1]Laboratory for Innovation in Microengineering (LiME), Department of Mechanical Engineering, University of Victoria, 3800 Finnerty Rd., Victoria, BC, Canada V8P 2C5

[2]Center for Advanced Materials and Related Technology (CAMTEC), University of Victoria, Victoria, Canada.

[3]Division of Neurosurgery, Department of Surgery, Vancouver General Hospital, University of British Columbia, Vancouver, BC, Canada.

[4]Departments of Human Anatomy and Cell Science, Rady Faculty of Health Science, University of Manitoba, Winnipeg, Canada

[5]The Biology of Breathing Theme, Children's Hospital Research Institute of Manitoba, University of Manitoba, Winnipeg, Canada.

[*]Corresponding author: Prof. M. Akbari (E-mail: makbari@uvic.ca)

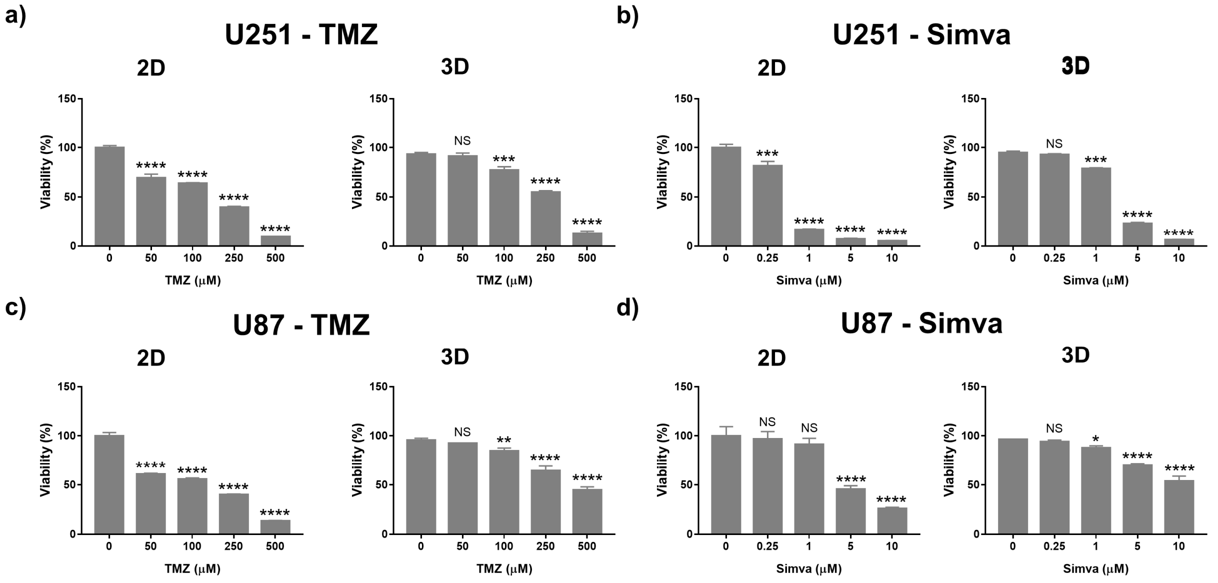

Figure S1 Statistical analysis for viability analysis of U251 and U87 cells after 72 hours of treatment with different concentrations of TMZ and Simva in 2D culture and GoC model. 2D and 3D viability were calculated using PrestoBlue and Live/Dead staining assays, respectively. Each set has been compared with its control goup. a) U251 cells treated with TMZ. b) U251 cells treated with Simva. c) U87 cells treated with TMZ. d) U87 cells treated with Simva. Data expressed as mean ± standard deviation (n=3). *P<0.05, **P<0.01, ***P<0.001, ****P<0.0001.

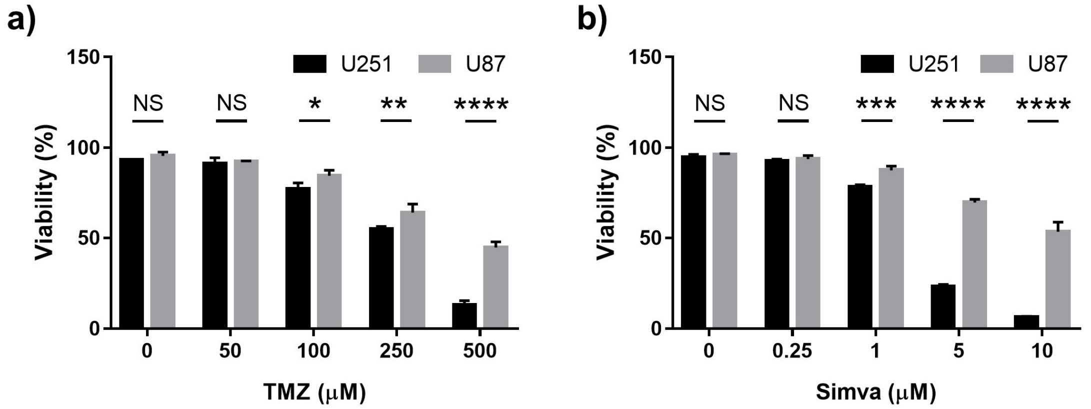

Figure S2 Statistical analysis for viability analysis of U251 and U87 cells in GoC model after 72 hours of treatment with different concentrations of TMZ and Simva. Viability was calculated using Live/Dead staining assay. a) Comparison of the response of U251 and U87 cells to different concentrations of TMZ. b) Comparison of the response of U251 and U87 cells to different concentrations of Simva. Data expressed as mean ± standard deviation (n=3). *P<0.05, **P<0.01, ***P<0.001, ****P<0.0001.

Bright field images of invasion analysis

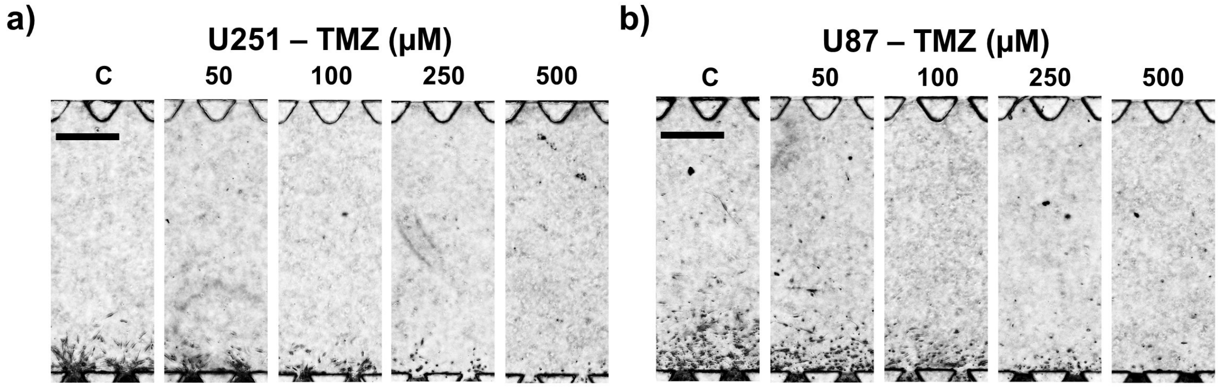

Figure S3. Bright field images of a) U251 and b) U87 cells invading to the side compartment under treatment with different concentration of temozolomide (0 – 500 μM).

Quantification of Vimentin marker

ImageJ was used for the quantification of the expression of vimentin in the cells under different treatments. Fig. S3a shows a sample of the immunofluorescence images taken from U87 cells treated with 5μM of simvastatin. The number of the DAPI-stained nuclei was counted as 8. For measuring the area of vimentin, first the scale was set in the software according to the scale bar from the microscopy image. Then, the green channel of the image was converted to grey scale color, as shown in Fig. S3b. The histogram thresholds of the image were set between the two values of 40 and 255, which was observed to be the best range considering all samples, and the background was set to white. The area of the black region was measured and divided to the number of the nuclei.

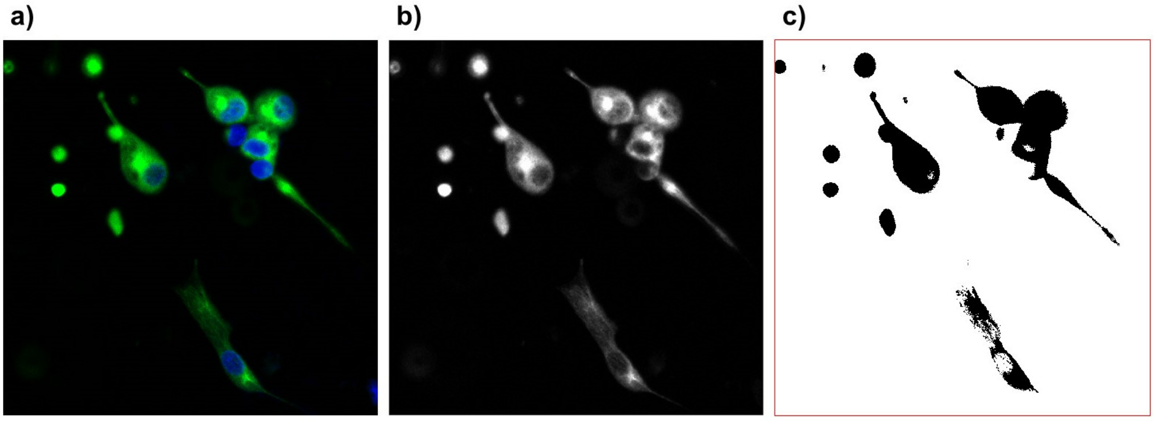

Figure S4 Quantification of vimentin. a) Immunofluorescence image if the cells, with nuclei stained in blue and vimenting stained in green. b) The green channel of the vimenting converted to grey scale. c) Adjusting the color threshold of the image such that the stained area is in blak and the background is in white.

$n = 8$ (the number of nuclei)
$A_V = 2129.28\ \mu m^2$ (calculated area of the vimentin)
Vimentin area per cell = $A_V / n = 2129.28\ \mu m^2 / 8 =$ **$266.16\ \mu m^2$ / cell**
